# Deep Learning-Based Caution Area Traffic Prediction with Automatic Identification System Sensor Data

**DOI:** 10.3390/s18093172

**Published:** 2018-09-19

**Authors:** Kwang-Il Kim, Keon Myung Lee

**Affiliations:** Department of Computer Science, Chungbuk National University, Cheongju 28644, Korea; kikim82@cbnu.ac.kr

**Keywords:** sensor data, deep learning, traffic prediction, automatic identification data sensor, convolution neural network, VTS

## Abstract

In a crowded harbor water area, it is a major concern to control ship traffic for assuring safety and maximizing the efficiency of port operations. Vessel Traffic Service (VTS) operators pay much attention to caution areas like ship route intersections or traffic congestion area in which there are some risks of ship collision. They want to control the traffic of the caution area at a proper level to lessen risk. Inertial ship movement makes swift changes in direction and speed difficult. It is hence important to predict future traffic of the caution area earlier on so as to get enough time for control actions on ship movements. In the harbor area, VTS stations collect a large volume of Automatic Identification Service (AIS) sensor data, which contain information about ship movement and ship attributes. This paper proposes a new deep neural network model called Ship Traffic Extraction Network (STENet) to predict the medium-term traffic and long-term traffic of the caution area. The STENet model is trained with AIS sensor data. The STENet model is organized into a hierarchical architecture in which the outputs of the movement and contextual feature extraction modules are concatenated and fed into a prediction module. The movement module extracts the features of overall ship movements with a convolutional neural network. The contextual modules consist of five separated fully-connected neural networks, each of which receives an associated attribute. The separation of feature extraction modules at the front phase helps extract the effective features by preventing unrelated attributes from crosstalking. To evaluate the performance of the proposed model, the developed model is applied to a real AIS sensor dataset, which has been collected over two years at a Korean port called Yeosu. In the experiments, four methods have been compared including two new methods: STENet and VGGNet-based models. For the real AIS sensor dataset, the proposed model has shown 50.65% relative performance improvement on average for the medium-term predictions and 57.65% improvement on average for the long-term predictions over the benchmark method, i.e., the SVR-based method.

## 1. Introduction

Maritime traffic has been increasing over the past decades with economic growth, and the scale of ports has been accordingly increased. Ship traffic routes, which are the sea lanes regularly used by ships to travel, are crowded with many inbound and outbound ships, especially in harbor sea areas. Ships can neither swiftly change their course, nor swiftly change their speed. Hence, careful monitoring and proactive control for ship traffic are important to avoid maritime accidents such as ship collisions, stranding, capsizing, and so on.

Ship traffic monitoring and control are difficult due to the following characteristics of maritime traffic in a harbor water area. First, no visible lanes are available on ship traffic routes. Second, ship traffic routes sometimes are merged, split or cross each other [1]. Third, inertial ship movement makes swift changes in direction and speed hard. Fourth, ship movement is affected not only by voyage schedule, but also by neighboring traffic and the environment [2].

For voyage and safety, many ships are equipped with various sensors like radar, GPS, Doppler log, Gyro compass, anemogram, and so on. In the water area, it is important for ships to broadcast their identifier and state information like position and movement to avoid maritime accidents and getting assistance. Those ships send out their AIS messages through an Automatic Identification System (AIS) transponder, which collects data from the installed onboard sensors [3]. A ship collects AIS messages aired by other ships to use them for safe navigation. Such collected data are called AIS sensor data, which contain various information about ship movement and attributes. The shore-side monitoring and controlling service stations, which are usually called VTSs (Vessel Traffic Service systems), collect AIS sensor data from their coverage and visualize the received data on the electronic navigational chart system for VTS operators. In addition, the VTSs provide ships with information about estimated traffic congestion and collision risks and enforce the regulations on navigation such as speed reduction, course change, and so on.

The on-duty VTS operators pay much attention to the caution area. A caution area means the crowded area at which many ship routes are merged, split or cross each other. In the caution area, there are some potential risks of ship collisions. To reduce collision risk, it is important to control the ship traffic at a proper level in the caution area [4]. To predict the traffic in the caution area at a future time point, we need some method to estimate the arrival time of a ship at the caution area. Experienced VTS operators have been trained in various similar situations for a long time and can predict future traffic in the caution area with some tools measuring the distance between two points. They take some control actions to maintain the caution area traffic at a proper level based on their estimation. This human-based approach is not good at medium-term and long-term predictions [5].

Real-time AIS sensor data are used firsthand for traffic surveillance at VTS stations. Once they are collected, they can be used later for some statistical analysis and machine learning tasks [6,7]. AIS sensor data are valuable assets with which new problem-solving methods might be developed.

Deep learning is a branch of machine learning that uses artificial neural networks with many layers, also known as deep neural networks, for approximating some function that fits well with the training data. The deep neural networks have the capability of automatically extracting and using relevant features appropriate for the target problem while being trained with the training data. There are various deep neural network models such as the Convolutional Neural Network (CNN), LSTM recurrent neural network, generative adversarial networks and capsule network [8]. Deep neural network models demand many training data because they have many parameters to be trained. Over the last ten years, deep learning techniques have demonstrated impressive performance improvement for difficult problems such as image and pattern classification, speech recognition, natural language processing tasks and signal processing tasks [9].

Various maritime traffic prediction methods have been developed [10,11,12,13,14,15,16,17,18,19,20,21]. Some methods are based on mathematical modeling [10,11]. It is difficult to develop satisfactory mathematical model-based traffic prediction because the traffic is not just governed by physical laws, but also by various regulations enforced in the harbor sea area. There are various machine learning-based prediction methods [12,13,14,15,16,17,18,19,20,21]. Deep neural networks are a powerful tool to attack some difficult problems like traffic prediction in a caution area. There are some works that have applied deep learning techniques in maritime applications. Nguyen et al. [22] proposed a recurrent neural network-based model to reconstruct trajectories, detect abnormal ship behaviors and identify ship type from AIS data. Gallego et al. [23] proposed a convolutional neural network-based method, which identified the type of ships in optical aerial images. We are yet unaware of any deep neural network-based maritime traffic density prediction methods.

This paper introduces a new deep neural model called STENet (Ship Traffic Extraction Network), which is trained with AIS sensor data and predicts the traffic of a caution area. The STENet model receives as the input both ship movement data and ship attribute data, which are extracted from AIS sensor data. The network produces as the output the predicted number of ships in the caution area at the future time points in 20, 30, 40 and 50 min.

The remainder of the paper is organized as follows: Section 2 presents some related works, and Section 3 explains the characteristics of AIS sensor data and describes how to prepare the AIS sensor data for a prediction model construction. Section 4 proposes the new deep neural network model STENet for the future traffic predictions in the caution area. Section 5 shows the experimental results of the proposed model for a real dataset for a Korean harbor. Finally, we draw the conclusions in Section 6.

## 2. Related Works

There are various works for maritime traffic prediction [12,13,14,15,16,17,18,19,20,21]. Perera et al. [12] proposed both a neural network-based method that detects and tracks multiple ships by using the radar data collected on the shore-side station and a Kalman filter-based method that predicts ship trajectories from current ship data. Xu et al. [13] proposed a short-term position prediction method that uses a multi-layered perceptron trained with ships’ position, course and speed data. Their trained model showed better accuracy in the ship movement prediction than the ship motion law-based method. Perera et al. (2010) [14] proposed a curvilinear motion model-based method to predict ocean-going ship trajectories and also proposed an extended Kalman filter-based algorithm to predict ship position, speed and acceleration.

There are some works to predict medium- and long-term maritime traffic using historical trajectories. Ristic et al. [15] proposed a method to predict individual ship’s trajectory at the future time points in 10, 32 and 70 min, which extracts representative trajectories by applying an adaptive kernel density estimation method to historical trajectory data. Mazzarella et al. [16] proposed a method to predict the ship trajectories and voyage times, which uses a particle filter-based simulation method and a velocity model. Their method uses the ship movement vector data extracted from AIS sensor data, but it does not take into account other neighboring ships’ traffic data.

Machine learning techniques have been applied for predicting maritime traffic [5,17,18,19], which train some machine learning models for traffic prediction with maritime data instead of developing man-made mathematical models. Xiao et al. [17] proposed a method to extract ship traffic patterns from ocean-going ship trajectories data with a DBSCAN-based clustering algorithm and to estimate short-term and long-term traffic by applying a kernel density estimation technique for the extracted ship traffic patterns. Kim et al. [5,18] proposed a method to predict ship position, speed and course in harbor areas, which trains a support vector regression model with ship trajectory data including the information about position and speed and then uses the trained regression model along with a dead reckoning estimation model. Zhang et al. [19] proposed a traffic prediction method for narrow water passage, which uses a support vector machine-based technique combined with a genetic algorithm for metaheuristic search. The method takes into account the ship trajectory data like position, speed and course, but does not consider other important factors such as ship destination, ship type and size and pilotage.

There are several neural network-based traffic prediction models that use both ship trajectory data and other traffic-related factors. Gan et al. [20] proposed a ship traffic estimation method for narrow water passage. The method first trains a neural network model with a hidden layer, which determines clusters of ship trajectory data along with ship’s speed, loading capacity, weight, maximum power and water level. Then, it uses the trained neural network model to choose the corresponding trajectory cluster to individual ship trajectories and then predicts the future traffic with the chosen trajectory clusters. Daranda [21] proposed a turning point-based path prediction method, which first identifies turning points with the DBSCAN-based clustering algorithm and then trains a multi-layered perceptron, which takes as input the ship information such as ship type, speed, course, length and position, and afterward outputs the next turning points. However, they proposed the prediction models, but did not present in detail the performance evaluation results on their methods. Although they tried to use both ship trajectory data and other traffic-related data, their neural network models were shallow networks, which are generally recognized to be inferior to recent deep neural networks.

We propose a deep neural network-based traffic prediction method for the caution area, which is trained with data consisting of ship movements and ship attributes. The proposed method is unique in that it uses a deep neural network model to predict the future traffic in the caution area with reference to all the available data about ship movements and attributes over the entire harbor area. To evaluate the performance of the proposed method, we conducted some experiments to apply it to a large real traffic dataset.

## 3. Automatic Identification System Sensor Data

### 3.1. Characteristics of Automatic Identification System Sensor Data

Ships with more than 300 gross tonnage with passengers are equipped with an AIS transponder device that broadcasts the ship’s dynamic, static and voyage information. Table 1 shows some data items in AIS messages and their broadcasting rates. An AIS message contains dynamic information about the ship’s movement such as position, speed, course and voyage status. The broadcasting rates of dynamic information messages depend on a ship’s movement status. For example, dynamic information is broadcasted every 3.3 s when a ship changes its course at a voyage speed of less than or equal to 14 knots and every 2 s when the voyage speed is greater than 23 knots. An AIS message conveys the ship’s static information such as ship name, call sign, ship type and ship specification. The static information does not change once the AIS device is installed on a ship. Voyage information can be conveyed in an AIS message, which is the semi-static data that do not change over one voyage from its departure port to its destination port. Voyage information includes freight information, ship draught, Estimated Time of Arrival (ETA), and so on. Because both ship’s static information and voyage information do not change over one voyage, where their AIS messages are broadcasted at a low frequency, e.g., every 6 min, AIS messages are sometimes delayed for a considerable amount of time due to the radio communication environment. The delay is caused by AIS device malfunctions, radio inferences with neighboring ships’ radio signals or geographic obstacles like islands [24]. AIS messages for dynamic information may sometimes contain invalid data due to the occasional malfunction of onboard AIS sensors, which are installed in a severe ship environment.

**Figure 1:**
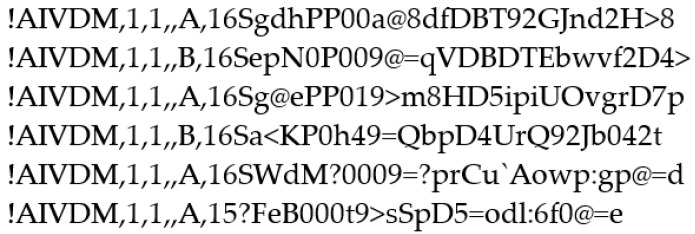
Examples of Automatic Identification Service (AIS) messages.

For ship traffic monitoring and controlling, VTS stations receive all AIS messages in their coverage areas with shore-based AIS devices. AIS messages contain the item of the message broadcast time, and hence, VTS stations can locate and keep track of the ships in their monitoring area. AIS messages occupy a time slot in their radio channel and are received in a stream data manner. Each message is encoded in the National Marine Electronics Association (NMEA) format. Figure 1 shows some examples of raw AIS messages in NMEA format. By parsing such raw AIS messages, we can extract the items shown in Table 1. Meanwhile, VTS stations save the received AIS messages in their storage for later use like safety and security analysis, forensic and maritime statistical analysis.

### 3.2. Ship Movement Data Preparation

We are interested in developing a machine learning-based method to predict the traffic in the entire harbor area rather than a method to predict the movement of individual ships. We need to have both ship movement data and other ship movement data obtained at synchronized time points to predict the traffic at specific future time points [25]. Ship movement data are crucial because they represent the movement vectors of ships, which are essential in predicting the future locations of ships.

**Figure 2:**
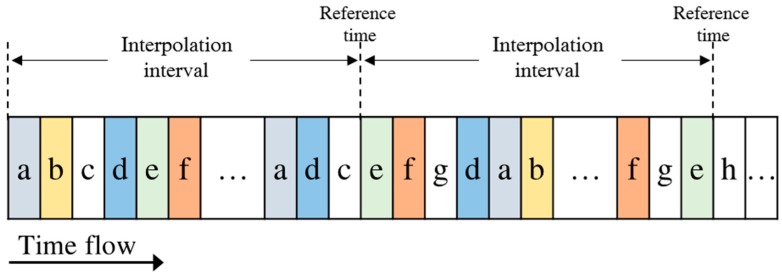
A sequence of a received AIS messages.

As mentioned in Section 3.1, the AIS messages are sometimes missing and their broadcasting intervals are different. Hence, the AIS data received at VTS stations are arranged in increasing order of their broadcasting time, as shown in Figure 2. To predict the traffic at a specific future time point, we are supposed to have movement data of the present time point. This means that all movement data should be synchronized at a specific time interval.

**Figure 3:**
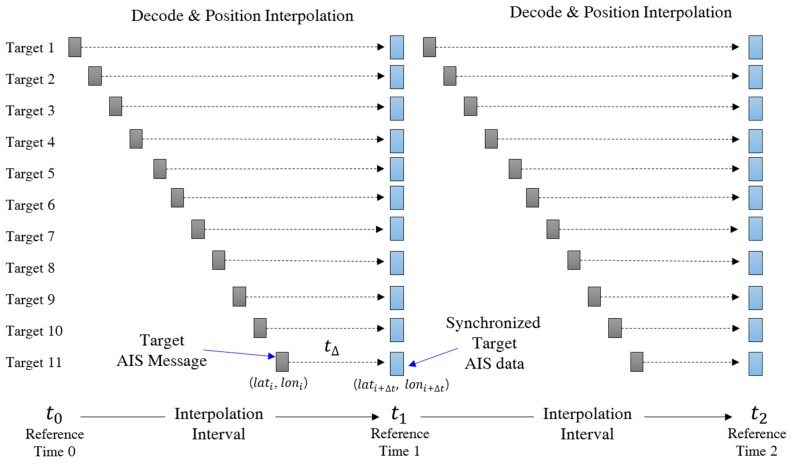
Synchronized AIS sensor data interpolation.

Figure 2 exemplifies some AIS messages sorted in increasing order of their broadcasting time where the letters in the boxes indicate the ship identifiers. We can see that the broadcasting times of received AIS messages are different from each other and the message arrival rates of ships are different from each other. To get movement data at specific intervals, we set an interpolation interval as shown in Figure 3 and interpolate movement data at the reference time points, which are the starting time of each interpolation interval.

The ship movement data at reference time points are generated by the interpolation method as follows [18]: First, we remove duplicated AIS messages for the same ship except the most recent message in each interpolation interval. Then, we apply an interpolation method to obtain the ship position at the reference time point. Let $t_{k}$ denote the k-th reference time point and $time\left(M_{i}\right)$ denote the broadcasting time of the message$\;M_{i}$. When an AIS message $M_{i}$ occurred in the interval between the k-th reference time and the k+1-th reference time, i.e., $t_{k}<time\left(M_{i}\;\right)\leq \;t_{k+1}$, the position [$lat_{i}$, $lon_{i}$] of the ship for $M_{i}$ is replaced with the position at time $t_{k+1}$, where the position is determined by the interpolation with the motion vector, i.e., course θ and speed v. Let $\Delta t=\;t_{k+1}-time\;\left(M_{i}\right)$. The following shows the interpolation equations for the new position at the reference time point [26].

$$\;lat_{i+\Delta t}=\arcsin\left[\sin\left(lat_{i}\right)\Delta \cos\frac{v\cdot \Delta t}{R}+\frac{\cos\left(m_{k}\right)}{\Delta t^{2}}\Delta \cos\left(lat_{i}\right)\Delta \sin\frac{V\cdot \Delta t}{R}\right],\;$$

(1) $$\;lon_{i+\Delta t}=lon_{i}+\arctan\left[\frac{\frac{\sin\left(m_{k}\right)}{\Delta t^{2}}\Delta \sin\frac{V\cdot \Delta t}{R}\Delta \cos\left(lat_{i}\right)}{\cos\frac{V\cdot \Delta t}{R}\Delta \sin\left(lat_{i}\right)\Delta \sin\left(lat_{i+\Delta t}\right)}\right]\;$$

Here, *R* indicates the radius of the earth, and $m_{k}$ denotes the angle between the x-axis and the course direction θ. $m_{k}$ is computed using the course direction as follows:

(2) $$\;m_{k}=\{\begin{array}{c} 90-\;\theta \;\;\;\;\;\;\;\;\;(0\leq \theta <90)\\ 90+\theta \;\;\;\;\;\;(90\leq \theta <180)\\ \begin{array}{c} 270-\theta \;(180\leq \theta <270)\\ 270+\theta \;(270\leq \theta <360) \end{array} \end{array}\;$$

The speed of a ship is measured by the unit knot (abbreviated as kt); one knot is the speed at which the ship travels one nautical mile for one hour. The ship speed is ranged over the interval from 0 knot–30 knots. The range of the course value is 0–359°. The course angles of 359° and 0° look very different, but they are very close. When we use the angle values in deep neural network models, we have to convert them into another representation, which allows the difference to be used for similarity computation. The new horizontal and vertical movement vectors ($V_{x}$, $V_{y}$) are computed as follows:

(3) $$V_{x}=\cos\left(m_{k}\right)\cdot v,\;V_{y}=\sin(m_{k})\cdot v$$

### 3.3. Association of Ship Attribute Data with AIS Data

In maritime traffic prediction, it is necessary to have ship movement data with the attributes such as position, velocity and course. In addition, there are other traffic-related factors such as ship length, ship type, ship destination, Pilot Onboard (POB) and Caution Area Estimated Time of Arrival (CAETA). Ship movement data, ship length and type information are directly obtained from AIS data. However, ship destination, POB and CAETA data are obtained by processing AIS sensor data. Ship length is extracted from a static information message of each ship, which is important in the traffic prediction because the longer a ship, the heavier it is, and hence, heavy ships show late responses for speed-up, speed-down or turning operations. The ship length affects the ship traffic prediction especially when a ship changes its speed or changes its course. For the convenience of handling in the prediction model, the length is normalized to have a value in the interval [0,1]. The normalized ship length is computing by dividing the ship length by the maximum ship length.

Ship type code in AIS messages ranges over the integers 0–99, i.e., there are 100 categories of ships. In the traffic prediction, the detailed categories of ships are not needed. Hence, we group 100 categories into three macro-categories, i.e., cargo ships, tanker ships and other ships. Cargo ships load their freight above the deck and have a low block coefficient, so that their navigation has a low influence from the under-water fluid. Therefore, they can travel fast, but take more time to change a course than other types of ships [27]. Tanker ships have the tankers under the deck, which are loaded with crude oil and chemical products. They have a high block coefficient, and hence, they are usually slower, but take a shorter time to change course than cargo ships. Other ships indicate small-sized ships like a pilot boat, operation boat, fishing boat, tug boat, and so on. They may travel on an arbitrary route and even cross the regular routes, if needed. They can easily change their course and can reduce their speed in a short time. Due to these characteristics, we regroup the 100 ship types into cargo ship, tanker ship and other ship.

The proposed method is concerned with predicting the future traffic in the caution area. Hence, the destination of ships is also an important factor to influence the caution area traffic. If the destination of a ship is located at the berth across the caution area, the ship should pass through the caution area. If a destination is near the caution area, the ship slows down its speed to come alongside the berth. The destination information in AIS sensor data is neither the berth’s name, nor the coordinate in the map, but a port name such as Port of South Louisiana and Busan Port. It is different to infer the destination berth from AIS sensor data. To estimate the destination berth for the training data, we examine AIS sensor data of each ship to see where the ship stops within a berth location range. Then, a ship destination is assigned with the ship location (*s.lat*, *s.lon*). Once the traffic prediction model is designed, then the destination of a ship is obtained by the VTS operator from the port management information system.

**Figure 4:**
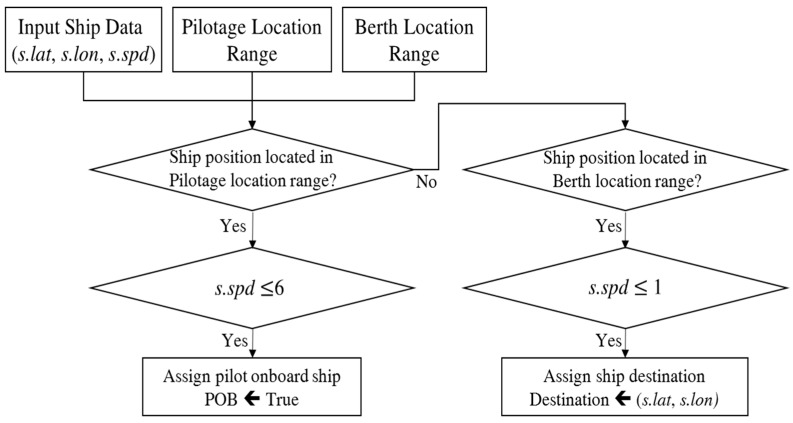
Pilot Onboard (POB) and destination information extraction procedure from AIS sensor data.

POB information indicates whether a pilot embarks or disembarks. On pilot embarkation or disembarkation, the ship slows down, and hence, POB information affects the traffic. When a ship comes into a port or departs from a port, then it slows down to 5~6 knots, which is the boarding speed if a pilot is scheduled to be on board. When constructing the training data, we examine the AIS sensor data of each ship to determine whether a pilot has gotten on board. We decided that a pilot has gotten on board if the ship slowed down to 4–6 knots in the pilot location range. Figure 4 shows the procedure used to extract POB and destination information of AIS sensor data for constructing the training data.

CAETA indicates the time taken for a ship *i* to travel from the current location ($s_{i}^{t}$*.lat*, $s_{i}^{t}$*.lon*) to the center of the caution area (c.lat, c.lon), at the present speed $s_{i}^{t}.spd$ at time *t*. Here, ($s_{i}^{t}$*.lat*, $s_{i}^{t}$*.lon*) and (c.lat, c.lon) indicate the pairs of the latitudinal and longitudinal positions for the ship’s location and the center of the caution area. CAETA can be computed by the following equation (Equation (4)):

(4) $$\;\mathrm{CAETA}=\frac{\sqrt{{\left(s_{i}^{t}.lat-c.lat\right)}^{2}+{\left(s_{i}^{t}.lon-c.lon\right)}^{2}}}{s_{i}^{t}.spd}\;$$

In the training data, CAETA is normalized to be in the range [0,1] by dividing CAETA by the expected maximum time. If CAETA is greater than the expected maximum time, its normalized value is set to one.

CAETA is useful information with which we can compute the estimated arrival time to the caution area under the condition that a ship maintains the present speed. Therefore, CAETA is taken as an input attribute for traffic density prediction in the caution area.

## 4. The Proposed Ship Traffic Prediction Method

This section presents a new deep neural network model, called the STENet (Ship Traffic Extraction Network) model, to predict the future ship traffic at the caution area, which is trained with AIS sensor data. The proposed deep neural network uses a Convolutional Neural Network (CNN) [28] as its subnetwork. CNN is representative of a deep neural network model, which takes multiple channels of two-dimensional data as input and repeatedly transforms them in convolution operations and optionally in the pooling operations. CNN extracts valuable features for problem solving from the input data by repetitive and consecutive convolution and pooling operations. The output of CNN is typically served as an input to a Fully-Connected Neural Network (FCNN), i.e., a multilayered perceptron. Section 4.1 presents how to represent the training data for the STENet model. Section 4.2 describes the STENet architecture in detail.

### 4.1. Encoding of AIS Data for the STENet Model

**Figure 5:**
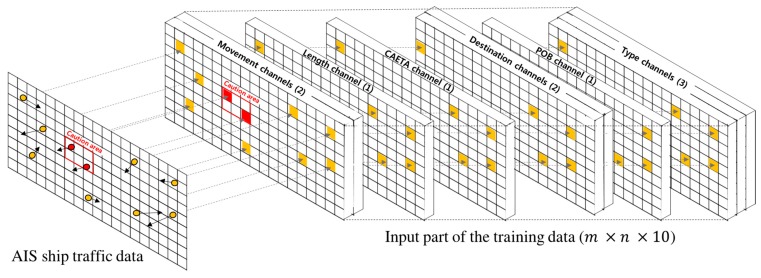
Input data encoding for the Ship Traffic Extraction Network (STENet) model.

The STENet model predicts the number of ships in the caution area at a medium-term and long-term future time from the harbor traffic status at the moment. The prediction model is trained with training data constructed from AIS sensor data. In Section 3.2 and Section 3.3, how the training data are constructed from AIS sensor data is presented. Because the information of ships is associated with geographical locations, the proposed method encodes the input part of the training data in 10 channels of a two-dimensional array, as shown in Figure 5. The training data for the STENet model consist of the movement vector, ship length, CAETA, destination, ship type and POB for input and the number of ships in the caution area at the designated future time point for output. The water area in a harbor is partitioned into an equi-sized m×m grid structure, each grid cell of which corresponds to a small water area and is associated with longitudinal and latitudinal coordinates. The grid size is designed enough to hold only one large ship with surrounding safety space, and it is assumed that at most one ship is located in a grid cell. In a two-dimensional array representation, each element corresponds to a grid cell in the water area. Hence, a grid cell is located by the index of the two-dimensional array. Each element in the two-dimensional array represents the information associated with the ship, if any, located at the corresponding grid cell.

Suppose that there are n ships in a harbor area at a time point t. The information $S^{t}$ for the ships is expressed as $S^{t}=\left\{s_{1}^{t},\;s_{2}^{t},\;\ldots ,s_{n}^{t}\right\}$, where $s_{i}^{t}$ indicates all information for the i-th ship at time point t. For $s_{i}^{t}$, its position index $\left(p_{i}^{t},\;q_{i}^{t}\right)$ at the two-dimensional arrays is found by comparing its position $\left(s_{i}^{t}.lat,\;s_{i}^{t}.lon\right)$ with the grid cell locations. The information of a ship is stored at the array element with the index.

The movement vector channels express the movement vectors of ships that are made of two components. The first channel contains the vector component $V_{x}\;$in the *x*-axis, and the second channel contains the vector component $V_{y}\;$in the *y*-axis. Hence, the movement vector channels are represented by an m×n×2 array. The movement vector channels at index $\left(p_{i}^{t},\;q_{i}^{t}\right)$ indicate the movement vector of the ship at the corresponding grid cell. The information of other ship attributes is also stored at the elements of the corresponding index $\left(p_{i}^{t},\;q_{i}^{t}\right)$ for their channels. Length, CAETA and POB are expressed in a single numerical value. Hence, each of them is stored in a single channel, i.e., m×n×1, respectively.

The destination information is represented by a vector D from the position of a ship to the destination coordinate. Suppose that ($s_{i}^{t}.d_{lat},\;s_{i}^{t}.d_{lon}$) denotes the coordinate of the destination of the i*-*th ship at time point t*.* Then, $D=\;\left(s_{i}^{t}.d_{lat},\;s_{i}^{t}.d_{lon}\right)-\left(s_{i}^{t}.lat,\;s_{i}^{t}.lon\right).$ The destination channels are made of two channels, one of which represents the vector component in the *x*-axis, and the other of which represents the vector component in the *y*-axis.

The proposed prediction model classifies ship type into one of cargo ship, tanker ship and other ship. Hence, the ship type information of a ship is encoded in an m×n×3 array, i.e., in three channels. Each channel corresponds to one type of ship, and the ship type information is expressed in one-hot encoding as shown in Table 2. In the table, we see that Ship A is a cargo ship located at the grid corresponding to the index (2,3).

On constructing the training dataset for the prediction model, the output is the predicted value. The STENet model is trained to predict the number of ships in the caution area at the future time points. For an input that consists of the values of six attributes at a specific time point t, its output is determined by computing the ships in the caution area for the interpolation interval at a future time t+Δ.

### 4.2. STENet Architecture

**Figure 6:**
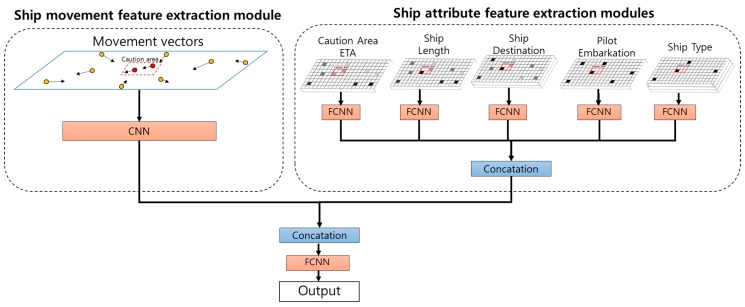
STENet architecture.

Figure 6 shows the proposed STENet architecture that is organized into a hierarchical model, of which the front part consists of a CNN module and five FCNN for feature extraction, of which the rear part is a fully-connected network to predict the future traffic using the extracted features. One CNN module is called the ship movement feature extraction module, which transforms the movement vector channels into a smaller feature map. The fully-connected network modules are called the ship attribute extraction modules, which take CAETA, ship length, destination, POB and channel type as the input and extract effective features from them. The outputs of both CNN module and the fully-connected modules of the front part are concatenated and flattened into one-dimensional data. The flattened data are fed into the rear-part of the fully-connected network, which produces the predicted number of ships in the caution area.

#### 4.2.1. Ship Movement Feature Extraction Module

**Figure 7:**
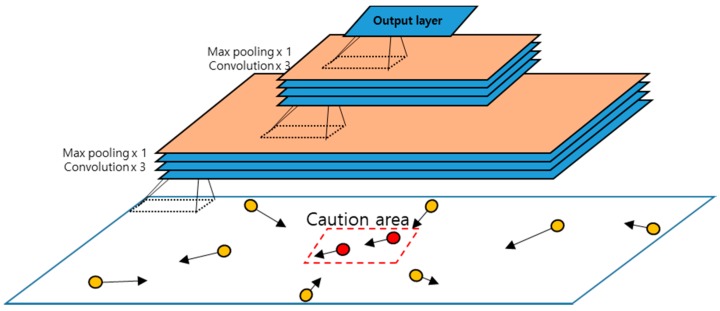
The CNN model for the ship movement feature extraction module.

The ship movement feature extraction module is implemented by a CNN model as shown in Figure 7. The CNN model is supposed to extract useful features for the spatial dependencies between overall ship positions and caution area. The input to the CNN model is the two channels of the ship movement vectors. The model is organized into the following architecture with seven layers:

Input(m,n,2)[-Conv-Conv-Conv-Maxpool]×2-Output(m/4,n/4,2)

Here, *Conv* indicates a convolution layer, which uses a kernel W to extract some features; *Maxpool* indicates the max pooling operation; and []×2 indicates the repetition of the subnetwork in the bracket []. In a CNN model, the kernels are learned from training data, while in conventional signal processing, the developers have to set the kernels appropriate fnnor a given task manually. The convolution is a transformation operator for an input with the kernel as follows: Suppose that input data $C^{n-1}\;$ is two-dimensional data of size, i.e., $C_{x}^{n-1}\times C_{y}^{n-1}$, and the kernel $W^{n}\;$ is a two-dimensional array of size, i.e., $K_{x}\times K_{y}$. Let * denote the convolution operation, and f is an activation function such as the sigmoid [29] or ReLU function [30]. When the convolution kernel $W^{n}\;$ is applied to the input $C^{n-1}$, then the output $C^{n}$ is computed as follows (here, $b^{n}$ is a bias term):

(5) $$\;C^{n}=f\left(\overset{C_{x}^{n-1}}{\underset{i=1}{\sum }}\overset{C_{y}^{n-1}}{\underset{j=1}{\sum }}C_{\left(i,j\right)}^{n-1}*W_{\left(i,j\right)}^{n}+b^{n}\right)\;$$

Max pooling indicates an operation to choose the maximum value for a specified region, which is usually a square region in its input. It plays the role of selecting the maximum feature values and of reducing the input into a smaller one.

In the CNN model for ship movement feature extraction, the convolution layers use a 3×3 convolution kernel with equi-padding, which makes the convolution result have the same dimension as its input, and max pooling operations are carried out with a 2×2 window. The input is given as an m×n×2 array, which represents the ship movement vectors expressed in two channels. On the other hand, the output is produced in an $\left(\frac{m}{4}\right)\times \left(\frac{n}{4}\right)\times 2\;$ array.

#### 4.2.2. Ship Attribute Feature Extraction Modules

The ship attribute extraction modules are made of five fully-connected networks, which take as the input the two-dimensional channels of CAETA, ship length, destination, POB and ship type, respectively. Each fully-connected network module has the following architecture with three layers, each of which has *p* nodes:

*Input*(*m*,*n*,*c*)-*FC*(*p*)-*FC*(*p*)-*FC*(*p*)-*Output*(*p*)

Here, *FC*(*p*) indicates a fully-connected layer with *p* nodes. For each fully-connected module, its input dimension is m×n×c, where *c* is the number of channels for the corresponding attribute, and the output dimension is p×1. The modules transform the ship attributes into compact feature vectors of dimension *p* that are effective for predicting future traffic in the caution area.

#### 4.2.3. Prediction Module

The prediction module consists of a fully-connected network with three hidden layers, and each of layers has 50, 30 and 10 nodes, respectively, and the output layer with a single node. The architecture has the outputs of the feature extraction modules as an input to the prediction module. It produces the predicted number of ships in the caution area in the medium-term and long-term futures. Moreover, it contains some additional operational layers for batch normalization and dropout to improve the performance.

In the fully-connected layers, each node is connected to all nodes of the very preceding layer. The output $N_{i}^{l+1}$ of the i-th node at layer *l* is computed as follows:

(6) $$\;N_{i}^{l+1}=f\left(\overset{n}{\underset{j=1}{\sum }}w_{i}^{l+1}N_{j}^{l}+b_{i}^{l+1}\right)\;$$

Here, $w_{\left(i,j\right)}^{l+1}$ is the connection weight between the i-th node at layer *l* and the j-th node at layer *l +* 1; $b_{i}^{l+1}$ is the bias term for the i-th node at layer *l +* 1; and f denotes the activation function. In FCNN, the exponential linear unit (ELU) function is used as the activation function, which is defined as follows [31]:

(7) $$\;f\left(x\right)=\{\begin{array}{c} x\;\;\;\;\;\;\;\;\;\;\;\;\;\;\;\;,\;x>0\\ \alpha \left(e^{x}-1\right),\;x\leq 0, \end{array}\;\;$$

Batch normalization is an operation that makes the input data to the next layer preserve a distribution of zero mean and unit variance. It is experimentally shown that the batch normalization is helpful for improving the performance and stability of a network.

To protect the fully-connected layers from overfitting the noisy or erroneous data, the dropout operations are used in the training phase [32]. When dropout is applied, randomly selected nodes are ignored by the network. The dropout operation is ignored in the inference phase.

### 4.3. Error Function and Performance Evaluation

STENet is trained to minimize the error function using a gradient descent-based training algorithm. As the error function, the network uses the Mean Absolute Percentage Error (MAPE), which is defined as follows [33]:

(8) $$\;MAPE=\left(\frac{1}{n}\overset{n}{\underset{j=1}{\sum }}\left|\frac{{\hat{y}}_{j}-y_{j}}{y_{j}}\right|\right)\times 100\;$$

where $y_{j}$ is the target output of the network and ${\hat{y}}_{j}$ is the predicted value by STENet. MAPE can measure the model with relative accuracy in the range of 0–100 in the training and validation phases.

## 5. Experiments

### 5.1. Data Preparation

**Figure 8:**
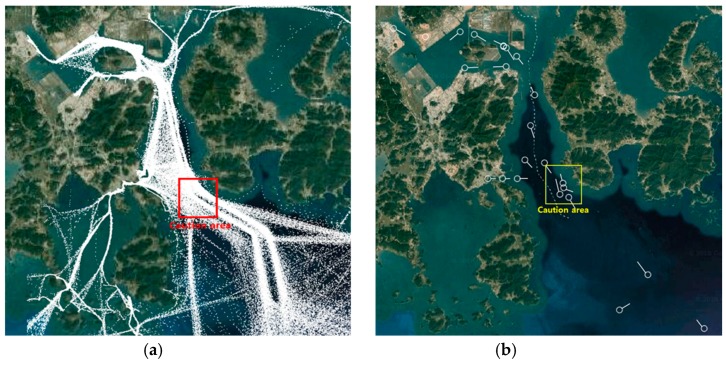
(**a**) The distribution of ship navigation trajectories and (**b**) the caution area.

To evaluate the performance of the proposed STENet model, we have used a real AIS sensor dataset collected over the two years (2015–2016) in Yeosu, which is a harbor located at the southern part of the Korean peninsula. According to the stability of their AIS sensor data, ships are categorized into either Class A or Class B, where ships to broadcast valid AIS messages are labeled with Class A and ships broadcasting invalid messages are labeled with Class B. The training data have been constructed from the AIS sensor data for Class A ships. Figure 8 shows the harbor water area for which AIS sensor data are collected by a VTS station. Figure 8a shows the distribution of ship trajectories over a day. In Figure 8b, the region indicated by a rectangle is the caution area for which we want to predict the future traffic. The region is the caution area because many inbound and outbound voyage routes are merged, crossed or split. It is important to control the number of ships in the region to be a manageable size for safety assurance. In the experiments, we implemented four prediction models to predict the future traffic at the caution area. We trained the models with the historical ship trajectory data constructed from AIS sensor data. The trained models are supposed to predict the caution area traffic at the future time points in 20, 30, 40 and 50 min with the real-time AIS sensor data over the entire harbor water area.

The dataset was constructed by the methods described in Section 3.2 and Section 3.3, where the interpolation interval was 10 s and the entire harbor water area was partitioned into a 100×100 grid. The size of merchant ships is on average 120 m, and in the normal situation, no such ships get closer to each other within a shorter distance than three-times the ship length to secure their safety. Hence the size of a grid cell was set 360 × 360$\;m^{2}$ in the experiments.

Hence, the harbor traffic at a specific time point was expressed in a 100×100×10 tensor. The synchronized data at the reference time points become the input part of the training data. The number of ships in the caution area at the future time points in 20, 30, 40 and 50 min become the output part of the training data. The number of ships ranged over the integer values from 0–9. If the number of ships was greater than 9, we clamped it to 9. For each future time point, a separate prediction model was trained. In total, four prediction models have been constructed. We acquired 25,470 distinct AIS trajectory data for the harbor over two years. Each trajectory consists of a sequence of AIS data from a harbor area limit to a berth in the harbor. There are 3 harbor area limits and 75 berths in the harbor; hence, there are 225 distinct routes. The training data for the prediction models, STENet and VGGNet, are generated by sampling the information of ship movement and attributes every 10 s. We get 8640 training data for a day. In a day, at many sampling times, there are no ships in the caution area, and hence, we selected the data in a way that too many data with zero ships in the caution area are included. Hence, the number of training data available is too small to train deep learning models like STENet and VGGNet.

Hence, we generated the training data by using the following augmentation method for the trajectory data. All the available trajectory data were partitioned into 225 groups according to their route. For each group, the occurrence probabilities of travels were computed over the hourly intervals in a day. The probability distributions show how frequently the corresponding trajectories happen in the routes. Every 10 s over a day, some groups are randomly selected according to the distributions. For each selected group, a trajectory is randomly selected from the group. Then, according to the trajectory, a ship is assumed to start at the starting point, i.e., harbor area limit or berth, of the trajectory, and the corresponding AIS data are observed. At the same time, a training data record is constructed with the 10 two-dimensional channels to describe the traffic situation at the moment as the input and the number of ships in the caution area as the output. With the augmentation method, we generated the training dataset of a size of 10,000,000. Then, 80% of the dataset was used to train the prediction model, and the remaining 20% was used to test the trained model.

### 5.2. Performance Evaluation

We compared the four prediction models: the Dead Reckoning (DR) model [16], Support Vector Regression (SVR) model [5], a new CNN-based model and the STENet model. In the DR model, it is assumed that the routes for ships are fixed in the harbor area and the ships travel over their route at their current speed. Then, after, the model determines the positions of ships at a specified time point by making them travel on their route at their current speed up to a prediction time point. Finally, the ships in the caution area are counted as the output. In the SVR model, the routes of ships are fixed, and the speeds at the segments of routes are trained by the SVR model. The future positions of ships are computed by making ships travel on the segments of their routes at the speeds for corresponding segments estimated by the SVR model. Similar to DR, the ships in the caution area are counted as the output.

Before developing the STENet model, we developed a new CNN-based prediction model that uses a simplified architecture of the VGGNet, which is generally known as a successful deep neural network model [34]. The VGGNet-based prediction model has the following layered architecture with 11 layers:

Input(100,100,10)-[*Conv*] × 2-Maxpool-[*Conv*] × 2-Maxpool-[*Conv*] × 3-Maxpool-[*Conv*] × 3-Maxpool-[*FC*]-Output(1)

Here, *Conv* indicates a 3×3 convolution layer; *Maxpool* indicates the max pooling operation with the 2×2 window; and *FC* indicates a fully-connected layer with a single output node. Later on, this model is denoted as the VGGNet model.

The same input data of size 100×100×10  prepared for the STENet model have been used for training the VGGNet prediction model. The STENet model has been trained with the prepared training dataset. The ADAM optimizer [35] was used for training the model with the learning rate η of 0.001 and hyperparameters $\beta_{1}=0.9$ and $\beta_{2}=0.999$. It is a gradient-descent method for function optimization that adjusts weights, each of which has its own learning rate. Each weight $w_{i}$ is tuned with the following update equation:

(9) $$\;w_{i}^{\left(t+1\right)}=w_{i}^{\left(t\right)}-\frac{\eta }{\sqrt{{\hat{v}}^{\left(t\right)}+\epsilon }}{\hat{m}}^{\left(t\right)}\;$$

Here, $w_{i}^{\left(t\right)}$ indicates the weight value of $w_{i}$ at time t, η is a hyperparameter and ϵ is a small positive value to avoid dividing by zero. Meanwhile, ${\hat{m}}^{\left(t\right)}$ and ${\hat{v}}^{\left(t\right)}$ are the estimates of the mean and variance of the gradients for $w_{i}$, which are adjusted by the parameters $\beta_{1}$ and $\beta_{2}$, respectively.

The prediction models have been developed for the future time points in 20, 30, 40 and 50 min. The 20- and 30-min future predictions are regarded as the medium-term predictions, and the 40- and 50-min future predications are regarded as the long-term predictions. A very long future prediction is not meaningful because most ships travel across the harbor within an hour.

The Mean Absolute Error (MAE) was used to evaluate the performance of the prediction models, which is defined as follows ($y_{j}$ is the true number of the ships in the caution area, and ${\hat{y}}_{j}$ is the estimated number of ships by the proposed prediction model):

(10) $$\;MAE=\overset{n}{\underset{j=1}{\sum }}\frac{\left|{\hat{y}}_{j}-y_{j}\right|}{n}\;$$

We compared the performance of the prediction models with respect to the performance of the SVR model. The relative performance improvement *RPI*(*M*) of a prediction model *M* is computed as follows (*MAE*(*SVR*) is the MAE of the SVR model):

(11) $$\;RPI\left(M\right)=\frac{MAE\left(SVR\right)-MAE\left(M\right)}{MAE\left(SVR\right)}\times 100\;$$

Table 3 shows the performance of the four prediction models with respect to the MAE and the Relative Performance Improvement (RPI).

The experimental results showed that the performance of the STENet model is improved on average by 50.65% for the medium-term predictions and 57.65% for the long-term predictions over the baseline model, i.e., the SVR-based model.

## 6. Conclusions

“Safety comes first” is at every corner of working environments. Maritime traffic shows different characteristics from ground traffic because of the difficulty in swiftly steering the ships on the water. Ship collision is a top-priority concern in ship monitoring and controlling services, especially in the caution area where many ship routes are merged, split or cross each other. The VTS operators are required to understand both ongoing situations and expected future situations in the caution area. We proposed a new deep neural network-based model called STENet for predicting future traffic in the caution area. The STENet model consists of the front-part feature extraction modules and the rear-part prediction module. All pieces of vessel information related to traffic are spatially encoded into two-dimensional channels from which features are separately extracted by a convolutional network and five fully-connected layers in the front-part of the model. The separated feature extraction helps the model performance improve by keeping unrelated attributes from crosstalking. The fully-connected layers of the rear-part of the model predict traffic density in the caution area by using those features together. It is also presented how to construct the training data from the AIS sensor data. The model predicts the medium- and long-term future traffic in the caution area. In the experiments on a real ship traffic dataset, the STENet model has shown superior performance to the reference model by a big margin.

The STENet model has the following advantages: First, it does not resort to a mathematical modeling for traffic predictions, but learns the prediction model from a large volume of AIS sensor data, which is easily collected on the VTS stations. Second, it can find excellent prediction models for medium- and long-term traffic predictions. Third, the encoding scheme for both ship movement and ship attribute data is effective at capturing the traffic characteristics. That can be justified by the fact that the STENet model has shown excellent performance on traffic prediction in the caution area. It is hence expected that if the output attribute is changed to other indicators like vessel collision risk or congestion rate, then STENet may find some good model for the indicator.

## Figures and Tables

**Table 1:** AIS sensor message information and update rates.

Type of Information	Information	Broadcasting Rate
Dynamic Information	Maritime Mobile Service Identity number (MMSI) Ship position Speed over ground Course over ground Navigational status Time Etc.	At anchor or moored (<3 kts): 3 min At anchor or moored (>3 kts): 10 s Ship 0–14 kts: 10 s Ship 0–14 kts and changing course: 3.3 s Ship 14–23 kts: 6 s Ship 14–23 kts and changing course: 2 s Ship >23 kts: 2 s
Static and Voyage-related Information	MMSI number Ship type Length and beam Estimated time of arrival Destination Etc.	Every 6 min and on request

**Table 2:** Examples of each channel and the layer index of the layer type.

Ship ID (Position Index)	Ship A (2,3)	Ship B (4,8)	Ship C (5,3)	Ship D (7,2)	Ship E (5,9)
Cargo ship channel	1	0	1	0	0
Tanker ship channel	0	0	0	1	0
Other ship channel	0	1	0	0	1

**Table 3:** Experiment results of ship traffic prediction.

			DR	SVR	VGGNet	STENet
Middle-term Prediction	20-min prediction	MAE (PRI)	1.004 (−11.1%)	0.904 (0%)	0.821 (9.2%)	0.415 (54.1%)
		SD	1.105	1.034	1.031	0.566
	30-min prediction	MAE (PRI)	1.541 (−25.1%)	1.232 (0%)	1.152 (6.5%)	0.651 (47.2%)
		SD	1.714	1.410	1.441	0.859
Long-term Prediction	40-min prediction	MAE (PRI)	2.510 (−46.8%)	1.710 (0%)	1.153 (32.6%)	0.717 (58.1%)
		SD	2.822	1.922	1.591	0.779
	50-min prediction	MAE (PRI)	3.5413 (−82.7%)	1.938 (0%)	1.436 (25.9%)	0.829 (57.2%)
		SD	3.673	2.057	1.754	1.077

** MAE: Mean Absolute Error, RPI: Relative Performance Improvement, SD: Standard Deviation, DR: Dead Reckoning.
